# Assessment of the further improved (*G'*/*G*)-expansion method and the extended tanh-method in probing exact solutions of nonlinear PDEs

**DOI:** 10.1186/2193-1801-2-326

**Published:** 2013-07-18

**Authors:** M Ali Akbar, Norhashidah Hj Mohd Ali, Syed Tauseef Mohyud-Din

**Affiliations:** Department of Applied Mathematics, University of Rajshahi, Rajshahi, Bangladesh; School of Mathematical Sciences, Universiti Sains Malaysia, Penang, Malaysia; Department of Mathematics, HITEC University Taxila Cantt Pakistan, Taxila, Pakistan

**Keywords:** The breaking soliton equation, Extended tanh-function method, Further improved (*G'*/*G*)-expansion method, Auxiliary equation, Traveling wave solutions

## Abstract

02.30.Jr, 05.45.Yv, 02.30.Ik

## Introduction

Nonlinear evolution equations play a significant role in various scientific and engineering fields, such as, optical fibers, solid state physics, fluid mechanics, plasma physics, chemical kinematics, chemical physics geochemistry etc. Nonlinear wave phenomena of diffusion, reaction, dispersion, dissipation, and convection are very important in nonlinear wave equations. In recent years, the exact solutions of nonlinear PDEs have been investigated by many researchers (see for example (Abdou 2007; Ablowitz & Clarkson 1991; Akbar et al. 2012a; Naher et al. 2012; Akbar et al. 2012b; Chen & Wang 2005; El-Wakil et al. 2010; Fan 2000; He & Wu 2006; Hirota 1971; Darvishi & Najafi 2012; Kawahara 1972; Kudryashov 1990; Kudryashov 1991; Kudryashov 2009; Liu et al. 2001; Lu et al. 2009; Lu 2005; Miura 1978; Parkes 2010; Rogers & Shadwick 1982; Bekir 2010; Wang & Zhang 2007; Wang & Zhou 2003; Wang & Li 2005a; Wang & Li 2005b; Wang et al. 2007; Wang et al. 2005; Ma et al. 2009; Wang et al. 2008; Wazwaz 2008a; Wazwaz 2008b; Inan 2010; Wazzan 2009; Yomba 2008; Naher et al. 2011; Yusufoglu & Bekir 2008; Zayed et al. 2004a; Zayed et al. 2004b; Zayed et al. 2007; Akbar & Ali 2011; Akbar et al. 2012c; Zhang et al. 2002; Akbar & Ali 2011; Akbar & Ali 2012; Zhang & Xia 2008; Zhang & Xia 2007; Zhang et al. 2008a; Zhang et al. 2008b)) who are concerned in nonlinear physical phenomena and many powerful and efficient methods have been offered by them. Among non-integrable nonlinear differential equations there is a wide class of equations that referred to as the partially integrable, because these equations become integrable for some values of their parameters. There are many different methods to look for the exact solutions of these equations. The most famous algorithms are the truncated Painleve expansion method (Kudryashov 1991), the Weierstrass elliptic function method (Kudryashov 1990), the tanh-function method (Abdou 2007; El-Wakil et al. 2010; Fan 2000; Wazwaz 2008b; Wazzan 2009; Zayed et al. 2004b; Zhang & Xia 2008) and the Jacobi elliptic function expansion method (Chen & Wang 2005; Liu et al. 2001; Lu 2005; Yusufoglu & Bekir 2008; Zayed et al. 2004a; Zayed et al. 2007). There are other methods which can be found in (Kawahara 1972; Wang & Zhang 2007; Wang & Zhou 2003; Wang & Li 2005a; Wang & Li 2005b; Wang et al. 2007; Wang et al. 2005). For integrable nonlinear differential equations, the inverse scattering transform method (Ablowitz & Clarkson 1991), the Hirota method (Hirota 1971), the truncated Painleve expansion method (Zhang et al. 2002), the Backlund transform method (Miura 1978; Rogers & Shadwick 1982) and the Exp-function method (Naher et al. 2012; He & Wu 2006; Naher et al. 2011; Akbar & Ali 2011; Akbar & Ali 2012) are used for searching the exact solutions.

Wang et al. (2008) introduced a direct and concise method, called the (*G'*/*G*)-expansion method to look for traveling wave solutions of nonlinear PDEs, where *G* = *G*(*ξ*) satisfies the second order linear ODE *G*″(*ξ*) + *λ G*′(*ξ*) + *μ G*(*ξ*) = 0; *λ* and *μ* are arbitrary constants. For additional references see the articles (Akbar et al. 2012a; Akbar et al. 2012b; El-Wakil et al. 2010; Parkes 2010; Akbar & Ali 2011; Akbar et al. 2012c; Zhang et al. 2008a; Zhang et al. 2008b).

In this article, we bring in an alternative approach, called a further improved (*G'*/*G*)-expansion method to find the exact traveling wave solutions of the breaking soliton equation, where *G* = *G*(*ξ*) satisfies the auxiliary ODE [*G*′(*ξ*)]^2^ = *p G*^2^(*ξ*) + *q G*^4^(*ξ*) + *r G*^6^(*ξ*); *p*, *q* and *r* are constants. Recently El-Wakil et al. (2010) and Parkes (2010) have shown that the extended tanh-function method proposed by Fan (2000) and the basic (*G'*/*G*)-expansion method proposed by Wang et al. (2008) are entirely equivalent in as much as they deliver exactly the same set of solutions to a given nonlinear evolution equation. This observation has also been pointed out recently by Kudryashov (2009). In this article, we assert even though the basic (*G'*/*G*)-expansion method is equivalent to the extended tanh-function method, the further improved (*G'*/*G*)-expansion method presented in this letter is not equivalent to the extended tanh-function method. The method projected in this article is varied to some extent from the extended (*G'*/*G*)-expansion method.

The objective of this article is to show that the further improved (*G'*/*G*)-expansion method and the celebrated extended tanh-function method are not identical. Further novel solutions are achieved via the offered further improved (*G'*/*G*)-expansion method. It has not been used by somebody previously. This approach will play an imperative role in constructing many exact traveling wave solutions for the nonlinear PDEs via the (2 + 1)-dimensional breaking soliton equation.

## The further improved (*G'*/*G*)-expansion method

Suppose we have the following nonlinear partial differential equation

1 $$F\left(u,u_{t},u_{x},u_{y},u_{\mathrm{tt}},u_{x\;t},u_{y\;t},\cdots \right)=0,$$

where *u* = *u*(*x*, *y*, *t*) is an unknown function, *F* is a polynomial in *u* = *u*(*x*, *y*, *t*) and its partial derivatives in which the highest order derivatives and the nonlinear terms are involved. In the following we give the main steps of the further improved (*G'*/*G*)-expansion method.

**Step 1**: The traveling wave variable,

2 $$\begin{array}{cc} u\;\left(x,y,t\right)=u\;(\xi ) & \xi =x+y-V\;t \end{array},$$

where *V* is the speed of the traveling wave, permits us to convert the Eq. (1) into an ODE in the form,

3 $$P\left(u,u\prime ,u\prime \prime ,u\prime \prime \prime ,\cdots \right)=0,$$

wherein $/=\frac{d}{\mathrm{d\xi}}$.

**Step 2**: Assume the solution of the Eq. (3) can be expressed by means of a polynomial in (*G'*/*G*) as follows:

4 $$u\left(\xi \right)=\sum_{i=0}^{n}\alpha_{i}{\left(\frac{G\prime }{G}\right)}^{i}$$

where *α*_*i*_(*i* = 1, 2, 3, ⋯) are constants provided *α*_*n*_ ≠ 0 and *G* = *G*(*ξ*) satisfies the following nonlinear auxiliary equation,

5 $${\left[G\prime \left(\xi \right)\right]}^{2}=p\;G^{2}\left(\xi \right)+q\;G^{4}\left(\xi \right)+r\;G^{6}\left(\xi \right),$$

Where *p*, *q* and *r* are random constants to be determined later (Table 1).

**Step 3**: In Eq. (4), *n* is a positive integer to be determined; typically this involves balancing the highest order nonlinear term(s) with the linear term(s) of the highest order come out in Eq. (3).

**Step 4**: Substituting Eq. (4), into Eq. (3) and utilizing Eq. (5), we obtain polynomials in *G*^*i*^(*ξ*) and *G'*(*ξ*) *G*^*i*^ (*ξ*) (*i* = 0, ± 1, ± 2, ± 3, ⋯). Vanishing each coefficient of the resulted polynomials to zero, yields a set of algebraic equations for *α*_*n*_, *p*, *q*, *r*, *V* and constant(s) of integration, if applicable. If the original evolution equation contains some arbitrary constant coefficients, these will, of course, also appear in the system of algebraic equations. Suppose with the aid of symbolic computation software such as Maple, the unknown constants *α*_*n*_, *p*, *q*, *r* and *V* can be found by solving these set of algebraic equations and substituting these values into Eq. (4), new and more general exact traveling wave solutions of the nonlinear partial differential Equation (1) can be found.

**Table 1:** **The general solutions of Eq. (5) are as follows (Yomba**^2008^**; Zhang** & **Xia**^2007^**)**

**No**	***G*** **(** ***ξ*** **)**	**No**	***G*** **(** ***ξ*** **)**
1	${\left[\frac{-p\;q\sec h^{2}\left(\sqrt{p}\;\xi \right)}{q^{2}-p\;r\;\left(1\pm \tanh\;\left(\sqrt{p}\xi \right)\right)}\right]}^{\frac{1}{2}}\mathrm{or}\;{\left[\frac{-p\;q\csc h^{2}\left(\sqrt{p}\;\xi \right)}{q^{2}-p\;r\;\left(1\pm \coth\;\left(\sqrt{p}\;\xi \right)\right)}\right]}^{\frac{1}{2}}$	6	${\left[\frac{-p\;\sec^{2}\left(\sqrt{-p}\xi \right)}{q\pm 2\;\sqrt{-p\;r}\tan\left(\sqrt{-p}\xi \right)}\right]}^{\frac{1}{2}}\mathrm{or}\;{\left[\frac{-p\;\csc^{2}\left(\sqrt{-p}\xi \right)}{q\pm 2\;\sqrt{-p\;r}\cot\left(\sqrt{-p}\;\xi \right)}\right]}^{\frac{1}{2}},\;p<0,\;r>0$
2	${\left[\frac{2\;p}{\pm \sqrt{\Delta }\cosh\;\left(2\sqrt{p}\xi \right)-q}\right]}^{\frac{1}{2}},p>0,\Delta >0$	7	${\left[\frac{p\;e^{\pm 2\;\sqrt{p}\xi }}{{\left(e^{\pm 2\;\sqrt{p}\xi }-4\;q\right)}^{2}-64\;p\;r}\right]}^{\frac{1}{2}},p>0$
3	${\left[\frac{2\;p}{\pm \sqrt{\Delta }\cos\;\left(2\sqrt{-p}\;\xi \right)-q}\right]}^{\frac{1}{2}}\mathrm{or}\;{\left[\frac{2\;p}{\pm \sqrt{\Delta }\sin\;\left(2\sqrt{-p}\;\xi \right)-q}\right]}^{\frac{1}{2}},p<0,\Delta >0$	8	${\left[-\frac{p}{q}\left(1\pm \tanh\;\left(\frac{1}{2}\sqrt{p}\xi \right)\right)\right]}^{\frac{1}{2}}\mathrm{or}{\left[-\frac{p}{q}\left(1\pm \coth\left(\frac{1}{2}\sqrt{p}\;\xi \right)\right)\right]}^{\frac{1}{2}},p>0,\Delta =0$
4	${\left[\frac{2\;p}{\pm \sqrt{-\Delta }\sinh\;\left(2\sqrt{p}\;\xi \right)-q}\right]}^{\frac{1}{2}},p>0,\Delta <0$	9	${\left[\frac{\pm p\;e^{\pm 2\;\sqrt{p}\xi }}{1-64\;p\;r\;e^{\pm 4\;\sqrt{p}\xi }}\right]}^{\frac{1}{2}},p>0,q=0$
5	${\left[\frac{-p\sec h^{2}\left(\sqrt{p}\;\xi \right)}{q\pm 2\;\sqrt{p\;r}\tanh\;\left(\sqrt{p}\xi \right)}\right]}^{\frac{1}{2}}\mathrm{or}{\left[\frac{p\csc h^{2}\left(\sqrt{p}\xi \right)}{q\pm 2\;\sqrt{p\;r}\coth\left(\sqrt{p}\;\xi \right)}\right]}^{\frac{1}{2}},p>0,\;r>0$	10	$\pm \frac{1}{\sqrt{q}\;\xi },p=0,r=0$

where Δ = *q*^2^ – *pr*.

## Application

In this section, we bring to bear the further improved (*G'*/*G*)-expansion method to the (2 + 1)-dimensional breaking soliton equation which is dreadfully important nonlinear evolution equations in mathematical physics and have been paid attention by a lot of researchers and the extended tanh-function method to compare the solutions obtained by the two methods.

### On solving the (2 + 1)-dimensional breaking soliton equation by the projected method

We start with the (2 + 1)-dimensional breaking soliton equation (Darvishi & Najafi 2012; Bekir 2010; Ma et al. 2009; Inan 2010) in the form,

6 $$u_{x\;t}-4\;u_{x\;y}u_{x}-2\;u_{x\;x}u_{y}-u_{x\;x\;x\;y}=0.$$

This equation was first introduced by Calogero and Degasperis in 1977. The breaking soliton equation describe the (2 + 1)-dimensional interaction of the Riemann wave propagation along the *y*-axis with a long wave propagation along *x*-axis (Ma et al. 2009). In the recent years, a considerable amount of research works on the breaking soliton equation have been accomplished. For example, its solitary wave solutions, periodic and multiple soliton solutions are found in (Inan 2010). Let us now solve the Eq. (6) by the proposed further improved (*G'*/*G*)-expansion method. To this end, we perceive that the traveling wave variable (2) permits us in converting Eq. (6) into an ODE and upon integration yields:

7 $$V\;u\prime +3\;{\left(u,\prime \right)}^{2}+u\prime \prime \prime =0$$

with zero constant of integration. Considering the homogeneous balance between the highest order derivative and the nonlinear term come out in Eq. (7), we deduce that *D*(*u*′)^2^ = *D*(*u*‴), where *D*(*u*′)^2^ stands for degree of (*u*′)^2^ and so on. This yield *n* = 1. Therefore, the solution (4) turns out to be

8 $$u\left(\xi \right)=\alpha_{1}\left(\frac{G\prime }{G}\right)+\alpha_{0}$$

Substituting (8) together with Eq. (5) into (7), we obtain the following polynomial equation in *G*:

9 $$\begin{array}{l} \alpha_{1}q\;\left(V+4\;p\right)\;G^{2}+\left(3\;\alpha_{1}^{2}q^{2}+32\;\alpha_{1}r\;p+2\;V\;\alpha_{1}r+6\;\alpha_{1}q^{2}\right)\;G^{4}\\ +12\;\alpha_{1}\;r\;q\;\left(\alpha_{1}+4\right)\;G^{6}+12\;\alpha_{1}\;r^{2}\left(\alpha_{1}+4\right)\;G^{8}=0 \end{array}$$

Setting each coefficient of the polynomial Eq. (9) to zero, we achieve a system of algebraic equations which can be solved by using the symbolic computation software such as Maple and obtain the following two sets of solutions:

The set 1.

10 $$\alpha_{1}=-4,\;\alpha_{0}=\alpha_{0},\;q=2\;\sqrt{p\;r},\;V=-4\;p,$$

where *α*_0_, *p* and *r* are arbitrary constants.

The set 2.

11 $$\alpha_{1}=-4,\;\alpha_{0}=\alpha_{0},\;q=0,\;V=-16p,$$

where *α*_0_, *p* and *r* are arbitrary constants.

Now for the set 1, we have the following solution:

12 $$u\left(\xi \right)=-4\;\left(\frac{G\prime }{G}\right)+\alpha_{0},$$

where

ξ=x+y+4pt.

According to the step 2 of section 2, we have the subsequent families of exact solutions:

**Family 1**. If *p* > 0, the solution of Eq. (5) has the form,

13 $$G\left(\xi \right)={\left[\frac{-p\;q\sec\;h^{2}\left(\sqrt{p}\;\xi \right)}{q^{2}-p\;r\;{\left(1\pm \tanh\;\left(\sqrt{p}\xi \right)\right)}^{2}}\right]}^{\frac{1}{2}}$$

or

14 $$G\left(\xi \right)={\left[\frac{-p\;q\csc h^{2}\left(\sqrt{p}\;\xi \right)}{q^{2}-p\;r\;{\left(1\pm \coth\;\left(\sqrt{p}\xi \right)\right)}^{2}}\right]}^{\frac{1}{2}}$$

In these cases we have the ratio,

15 $$\frac{G\prime }{G}=\frac{\sqrt{p}\left\{\;q^{2}\sinh\;\left(\sqrt{p}\;\xi \right)\cosh\left(\sqrt{p}\;\xi \right)-2p\;r\sinh\;\left(\sqrt{p}\;\xi \right)\cosh\left(\sqrt{p}\;\xi \right)\mp 2\;p\;r\;\cosh^{2}\left(\sqrt{p}\;\xi \right)\pm p\;r\right\}}{-q^{2}\cosh^{2}\left(\;\sqrt{p}\;\xi \right)+2\;p\;r\;\cosh^{2}\left(\sqrt{p}\;\xi \right)\pm 2p\;r\sinh\;\left(\sqrt{p}\;\xi \right)\cosh\;\left(\sqrt{p}\;\xi \right)-p\;r}$$

and

16 $$\frac{G\prime }{G}=\frac{\sqrt{p}\left\{q^{2}\sinh\left(\sqrt{p}\;\xi \right)\cosh\;\left(\sqrt{p}\;\xi \right)-2p\;r\sinh\;\left(\sqrt{p}\;\xi \right)\cosh\;\left(\sqrt{p}\;\xi \right)\mp 2\;p\;r\;\cosh^{2}\left(\sqrt{p}\;\xi \right)\pm p\;r\right\}}{-q^{2}\cosh^{2}\left(\;\sqrt{p}\;\xi \right)+2\;p\;r\;\cosh^{2}\left(\sqrt{p}\;\xi \right)\pm 2p\;r\sinh\;\left(\sqrt{p}\;\xi \right)\cosh\;\left(\sqrt{p}\;\xi \right)+q^{2}-p\;r}$$

respectively.

Since $q=2\;\sqrt{p\;r}$, subsequently, we obtain the following traveling wave solutions,

17 $$u\left(\xi \right)=\mp 4\;\sqrt{p}\pm \frac{8\sqrt{p}\sec h^{2}\left(2\;\sqrt{p}\;\xi \right)}{\sec h^{2}\left(2\;\sqrt{p}\;\xi \right)\mp 2\tanh\left(2\;\sqrt{p}\;\xi \right)+2}+\alpha_{0}$$

and

18 $$u\left(\xi \right)=4\;\sqrt{p}-\frac{8\sqrt{p}\sec h^{2}\left(2\;\sqrt{p}\;\xi \right)}{\;3\sec h^{2}\left(2\;\sqrt{p}\;\xi \right)+2\tanh\left(2\;\sqrt{p}\;\xi \right)\mp 2}+\alpha_{0},$$

where

ξ=x+y+4pt

**Family 2**. If *p* > 0, *r* > 0, the solution of Eq. (5) has the form,

19 $$\begin{array}{c} G\left(\xi \right)={\left[\frac{-p\sec h^{2}\left(\sqrt{p}\;\xi \right)}{q\pm 2\sqrt{p\;r}\tanh\left(\sqrt{p}\xi \right)}\right]}^{\frac{1}{2}}\;\mathrm{or}\;G(\xi )\\ ={\left[\frac{p\csc h^{2}\left(\sqrt{p}\;\xi \right)}{q\pm 2\sqrt{p\;r}\coth\left(\sqrt{p}\xi \right)}\right]}^{\frac{1}{2}}. \end{array}$$

Then we have the ratio,

$$\frac{G\prime }{G}=\frac{\sqrt{p}\;\left\{\pm q\sinh\left(\sqrt{p}\;\xi \right)\cosh\;\left(\sqrt{p}\;\xi \right)\mp 2\;\sqrt{p\;r}\;\cosh^{2}\left(\sqrt{p}\;\xi \right)\mp \sqrt{p\;r}\right\}}{\cosh\;\left(\sqrt{p}\;\xi \right)\;\left\{\;q\cosh\;\left(\sqrt{p}\;\xi \right)\pm 2\;\sqrt{p\;r}\sinh\;\left(\sqrt{p}\;\xi \right)\right\}}$$

or

20 $$\frac{G\prime }{G}=\frac{-\sqrt{p}\left\{\pm q\sinh\;\left(\sqrt{p}\;\xi \right)\cosh\;\left(\sqrt{p}\;\xi \right)\pm 2\;\sqrt{p\;r}\;\cosh^{2}\left(\sqrt{p}\;\xi \right)\mp \sqrt{p\;r}\;\right\}}{\sinh\;\left(\sqrt{p}\;\xi \right)\;\left\{q\sinh\;\left(\sqrt{p}\;\xi \right)\pm 2\;\sqrt{p\;r}\cosh\;\left(\sqrt{p}\;\xi \right)\right\}}.$$

Since $q=2\;\sqrt{p\;r}$, subsequently, we obtain the following traveling wave solutions:

$$u\;\left(\xi \right)=\pm 2\;\sqrt{p}+2\;\sqrt{p}\tanh\;\left(\sqrt{p}\;\xi \right)+\alpha_{0}\;$$

or

21 $$u\left(\xi \right)=\pm 2\;\sqrt{p}+2\;\sqrt{p}\coth\;\left(\sqrt{p}\;\xi \right)+\alpha_{0}$$

where

ξ=x+y+4pt

**Family 3**. If *p* < 0, *r* > 0, the solution of Eq. (5) has the form,

$$G\left(\xi \right)={\left[\frac{-p\;\sec^{2}\left(\sqrt{-p}\;\xi \right)}{q\pm 2\sqrt{-p\;r}\tan\left(\sqrt{-p}\xi \right)}\right]}^{\frac{1}{2}}$$

or

22 $$G\left(\xi \right)={\left[\frac{-p\;\csc^{2}\left(\sqrt{-p}\;\xi \right)}{q\pm 2\;\sqrt{-p\;r}\cot\;\left(\sqrt{-p}\;\xi \right)}\right]}^{\frac{1}{2}}.$$

Then we have the ratio

$$\frac{G\prime }{G}=\frac{\sqrt{-p}\;\left\{\sqrt{-p\;r}-2\;\sqrt{-p\;r}\cos^{2}\left(\sqrt{-p}\;\xi \right)\pm q\sin\;\left(\sqrt{-p}\;\xi \right)\cos\;\left(\sqrt{-p}\;\xi \right)\right\}}{\cos\;\left(\sqrt{-p}\;\xi \right)\;\left\{\;2\;\sqrt{-p\;r}\sin\;(\sqrt{-p}\;\xi \pm q\cos\left(\sqrt{-p}\;\xi \right)\right\}}$$

or

23 $$\frac{G\prime }{G}=\frac{\sqrt{-p}\left\{\sqrt{-p\;r}\mp 2\;\sqrt{-p\;r}\;\cos^{2}\left(\sqrt{-p}\;\xi \right)\mp q\sin\;\left(\sqrt{-p}\;\xi \right)\cos\;\left(\sqrt{-p}\;\xi \right)\right\}}{\sin\;\left(\sqrt{-p}\;\xi \right)\;\left\{2\;\sqrt{-p\;r}\cos\;\left(\sqrt{-p}\;\xi \right)\pm q\sin\;\left(\sqrt{-p}\;\xi \right)\right\}}.$$

Since $q=2\;\sqrt{p\;r}$, subsequently, we obtain the following traveling wave solutions:

$$u\left(\xi \right)=\frac{-4\;\sqrt{-p}\;\left\{\tan\;\left(2\;\sqrt{-p}\;\xi \right)\mp 1\;\right\}}{1\pm \tan\left(2\;\sqrt{-p}\;\xi \right)+\sec\;\left(2\;\sqrt{-p}\;\xi \right)}+\alpha_{0}$$

or

24 $$u\left(\xi \right)=\frac{-4\;\sqrt{-p}\;\left\{\tan\;\left(2\;\sqrt{-p}\;\xi \right)\pm 1\right\}}{1\mp \tan\left(2\;\sqrt{-p}\;\xi \right)-\sec\;\left(2\;\sqrt{-p}\;\xi \right)}+\alpha_{0},$$

where

ξ=x+y+4pt.

**Family 4**. If *p* > 0, Δ = 0, the solution of Eq. (5) has the form,

$$G\left(\xi \right)={\left[-\frac{p}{q}\left\{1\pm \tanh\;\left(\frac{1}{2}\sqrt{p}\xi \right)\right\}\right]}^{\frac{1}{2}}$$

or

25 $$G\left(\xi \right)={\left[-\frac{p}{q}\;\left\{1\pm \coth\;\left(\frac{1}{2}\sqrt{p}\;\xi \right)\right\}\right]}^{\frac{1}{2}}$$

Then we have the ratio

$$\frac{G\prime }{G}=\frac{\sqrt{p}}{4}\left\{\pm 1-\tanh\;\left(\frac{1}{2}\sqrt{p}\;\xi \right)\right\}$$

or

26 $$\frac{G\prime }{G}=\frac{\sqrt{p}}{4}\;\left\{\pm 1-\coth\;\left(\frac{1}{2}\sqrt{p}\;\xi \right)\right\}$$

Subsequently, we obtain the following traveling wave solutions:

$$u\left(\xi \right)=-\sqrt{p}\;\left\{\pm 1-\tanh\;\left(\frac{1}{2}\sqrt{p}\;\xi \right)\right\}+\alpha_{0}$$

or

27 $$u\left(\xi \right)=-\sqrt{p}\;\left\{\pm 1-\coth\left(\frac{1}{2}\sqrt{p}\;\xi \right)\right\}+\alpha_{0},$$

where

ξ=x+y+4pt

**Family 5**. If p > 0, the solution of Eq. (5) has the form

28 $$G\left(\xi \right)={\left\{\frac{p\;e^{\pm 2\;\sqrt{p}\;\xi }}{{\left(e^{\pm 2\;\sqrt{p}\xi }-4\;q\right)}^{2}-64\;p\;r}\right\}}^{\frac{1}{2}}$$

Then we have the ratio

29 $$\frac{G\prime }{G}=\frac{\sqrt{p}\;\left\{\;e^{\pm 4\;\sqrt{p}\;\xi }-16\;q^{2}+64\;p\;r\right\}}{\mp e^{\pm 4\;\sqrt{p}\;\xi }\pm 8\;q\;e^{\pm 2\;\sqrt{p}\;\xi }\mp 16\;q^{2}\pm 64\;p\;r}$$

Since $q=2\;\sqrt{p\;r}$, subsequently, we obtain the following traveling wave solutions:

30 $$u\left(\xi \right)=\frac{-4\;\sqrt{p}\;e^{\pm 2\;\sqrt{p}\;\xi }}{\pm 16\;\sqrt{p\;r}\mp e^{\pm 2\;\sqrt{p}\;\xi }}+\alpha_{0}$$

where

ξ=x+y+4pt

For the set 2, we have the following solution:

31 $$u\left(\xi \right)=-4\;\left(\frac{G\prime }{G}\right)+\alpha_{0}$$

where

ξ=x+y+16pt.

According to the step 2 of section 2, we obtain the subsequent families of exact solutions:

Cohort 1. If *p* > 0, Δ > 0, the solution of Eq. (5) has the form,

32 $$G\left(\xi \right)={\left[\frac{2\;p}{\pm \sqrt{\Delta }\cosh\;\left(2\;\sqrt{p}\xi \right)-q}\right]}^{\frac{1}{2}}$$

Since *q* = 0, then *r* < 0. In this case we have the ratio,

33 $$\frac{G\prime }{G}=-\sqrt{p}\tanh\;\left(2\;\sqrt{p}\;\xi \right)$$

Therefore, we obtainthe following traveling wave solution,

34 $$u\left(\xi \right)=4\;\sqrt{p}\tanh\;\left(2\;\sqrt{p}\;\xi \right)+\alpha_{0},$$

where

ξ=x+y+16pt.

Cohort 2. If *p* > 0, Δ < 0, the solution of Eq. (5) has the form

35 $$G\left(\xi \right)={\left[\frac{2\;p}{\pm \sqrt{-\Delta }\sinh\;\left(2\;\sqrt{p}\xi \right)-q}\right]}^{\frac{1}{2}}$$

Since *q* = 0, then *r* > 0. In this case we have the ratio,

36 $$\frac{G\prime }{G}=-\sqrt{p}\coth\;\left(2\;\sqrt{p}\;\xi \right).$$

Therefore, we obtain the following traveling wave solutions:

37 $$u\left(\xi \right)=4\;\sqrt{p}\coth\;\left(2\;\sqrt{p}\;\xi \right)+\alpha_{0},$$

where

ξ=x+y+16pt.

Cohort 3. If *p* < 0, Δ > 0, the solutions of Eq. (5) has the form,

$$G\left(\xi \right)={\left[\frac{2\;p}{\pm \sqrt{\Delta }\cos\;\left(2\;\sqrt{-p}\;\xi \right)-q}\right]}^{\frac{1}{2}}$$

or

38 $$G\left(\xi \right)={\left[\frac{2\;p}{\pm \sqrt{\Delta }\sin\;\left(2\;\sqrt{-p}\;\xi \right)-q}\right]}^{\frac{1}{2}}$$

Since *q* = 0, then *r* > 0. Thus we have the ratio,

$$\frac{G\prime }{G}=\sqrt{-p}\tan\;\left(2\;\sqrt{-p}\;\xi \right)$$

or

39 $$\frac{G\prime }{G}=-\sqrt{-p}\cot\;\left(2\;\sqrt{-p}\;\xi \right)$$

Therefore, we obtain the following traveling wave solutions:

$$u\left(\xi \right)=-4\sqrt{-p}\tan\;\left(2\;\sqrt{-p}\;\xi \right)+\alpha_{0}$$

or

40 $$u\left(\xi \right)=-4\;\sqrt{-p}\cot\;\left(2\;\sqrt{-p}\;\xi \right)+\alpha_{0}$$

where

ξ=x+y+16pt.

Cohort 4. If *p* > 0, *r* > 0, the solutions of Eq. (5) has the form,

$$G\left(\xi \right)={\left[\frac{-p\sec h^{2}\left(\sqrt{p}\;\xi \right)}{q\pm 2\sqrt{p\;r}\tanh\;\left(\sqrt{p}\xi \right)}\right]}^{\frac{1}{2}}$$

or

41 $$G\left(\xi \right)={\left[\frac{p\csc h^{2}\left(\sqrt{p}\;\xi \right)}{q\pm 2\;\sqrt{p\;r}\coth\;\left(\sqrt{p}\;\xi \right)}\right]}^{\frac{1}{2}}.$$

Since *q* = 0, we have the ratio,

42 $$\frac{G\prime }{G}=-\frac{1}{2}\sqrt{p}\;\left[\tanh\;\left(\sqrt{p}\;\xi \right)+\coth\;\left(\sqrt{p}\;\xi \right)\right]$$

Therefore, we obtain the following traveling wave solution:

43 $$u\left(\xi \right)=2\;\sqrt{p}\;\left\{\tanh\;\left(\sqrt{p}\;\xi \right)+\coth\;\left(\sqrt{p}\;\xi \right)\right\}+\alpha_{0}$$

where

ξ=x+y+16pt

Cohort 5. If p < 0, *r* > 0, the solutions of Eq. (5) has the form

$$G\left(\xi \right)={\left[\frac{-p\;\sec^{2}\left(\sqrt{-p}\;\xi \right)}{q\pm 2\sqrt{-p\;r}\tan\;\left(\sqrt{-p}\xi \right)}\right]}^{\frac{1}{2}}$$

or

44 $$G\left(\xi \right)={\left[\frac{-p\;\csc^{2}\left(\sqrt{-p}\;\xi \right)}{q\pm 2\;\sqrt{-p\;r}\cot\;\left(\sqrt{-p}\;\xi \right)}\right]}^{\frac{1}{2}}$$

Since *q* = 0, then we have the ratio,

45 $$\frac{G\prime }{G}=-\frac{1}{2}\sqrt{-p}\;\left[\cot\;\left(\sqrt{-p}\;\xi \right)-\tan\;\left(\sqrt{-p}\;\xi \right)\right].$$

Therefore, we obtain the following traveling wave solutions:

46 $$u\left(\xi \right)=2\;\sqrt{-p}\;\left\{\cot\;\left(\sqrt{-p}\;\xi \right)-\tan\;\left(\sqrt{-p}\;\xi \right)\right\}+\alpha_{0},$$

where

ξ=x+y+16pt.

Cohort 6. If *p* > 0, *q* = 0, the solution of Eq. (5) has the form,

47 $$G\left(\xi \right)={\left[\frac{\pm p\;e^{\pm 2\;\sqrt{p\;\xi }}}{1-64\;p\;r\;e^{\pm 4\;\sqrt{p\;\xi }}}\right]}^{\frac{1}{2}}$$

Then we have the ratio,

48 $$\frac{G\prime }{G}=\pm \frac{1}{8\;\sqrt{r}}\coth\;\left(\frac{\pm \xi }{4\;\sqrt{r}}\right),$$

where 64 *pr* = 1.

Therefore, we have the solution:

49 $$u\left(\xi \right)=\mp \frac{1}{2\;\sqrt{r}}\coth\;\left(\frac{\pm \xi }{4\;\sqrt{r}}\right)+\alpha_{0},$$

where

ξ=x+y+16pt.

Cohort 7. If *p* = 0, *r* = 0, then the solution of Eq. (5) has the form,

50 $$G\left(\xi \right)=\pm \frac{1}{\sqrt{q}\;\xi }.$$

Then we have the ratio,

51 $$\frac{G\prime }{G}=-\frac{1}{\xi }+\alpha_{0}.$$

Therefore, we have the solution:

52 $$u\left(\xi \right)=\frac{4}{\xi }+\alpha_{0},$$

where

ξ=x+y+16pt.

These are the exact solutions of the breaking soliton equation obtained by the further improved (*G'*/*G*)-expansion method.

### On solving the breaking soliton equation by the extended tanh-function method

With reference to the well-known extended tanh-function method (Abdou 2007; El-Wakil et al. 2010; Fan 2000; Wazwaz 2008b; Wazzan 2009; Zayed et al. 2004b; Zhang & Xia 2008), the solution of the breaking soliton Eq. (6) can be written in the form,

53 $$u\left(\xi \right)=\alpha_{1}\;\phi \left(\xi \right)+\alpha_{0},$$

where *ϕ*(*ξ*) satisfy the Riccati equation

54 $$\phi \prime \left(\xi \right)=A+\phi^{2}\left(\xi \right).$$

The Riccati Eq. (54) has the following solutions:

(i) If *A* < 0, then
  $$\phi \left(\xi \right)=-\sqrt{-A}\tanh\;\left(\sqrt{-A}\;\xi \right)$$
  or
  55 $$\phi \left(\xi \right)=-\sqrt{-A}\coth\;\left(\sqrt{-A}\;\xi \right).$$
(ii) If *A* > 0, then
  56 $$\phi \left(\xi \right)=\sqrt{A}\tan\;\left(\sqrt{A}\;\xi \right)\;\mathrm{or}\;\phi \left(\xi \right)=-\sqrt{A}\cot\;\left(\sqrt{A}\;\xi \right)$$
(iii) If *A* = 0, then
  57 $$\phi \left(\xi \right)=-\frac{1}{\xi }.$$

Substituting (53) together with (54) into (7), we obtain the following polynomial equation in *φ*:

58 $$\begin{array}{c} 3\;\alpha_{1}\;\left(\alpha_{1}+2\;\right)\;\varphi^{4}\left(\xi \right)+\alpha_{1}\;\left(V+8\;A+6\;\alpha_{1}A\right)\;\varphi^{2}\left(\xi \right)\\ \;+\alpha_{1}\;A\;\left(V+2\;A+3\;\alpha_{1}\;A\right)=0 \end{array}$$

Equating the coefficients of this polynomial to zero and solving the set of algebraic equations with the aid of Maple, we obtain the subsequent solution:

59 $$\alpha_{1}=-2,\;\alpha_{0}=\alpha_{0},\;V=4\;A$$

where *α*_0_ and *A* are arbitrary constants.

Accordingly the exact solutions of Eq. (6) have the following forms:

When *A* < 0, the solution takes the form,

$$\phi \left(\xi \right)=2\sqrt{-A}\tanh\;\left(\sqrt{-A}\;\xi \right)+\alpha_{0}\;$$

or

60 $$\phi \left(\xi \right)=2\sqrt{-A}\coth\;\left(\sqrt{-A}\;\xi \right)+\alpha_{0},$$

where

ξ=x+y−4At.

When *A* > 0, the solution takes the form,

$$\phi \left(\xi \right)=-2\;\sqrt{A}\tan\;\left(\sqrt{A}\;\xi \right)+\alpha_{0}\;$$

or

61 $$\phi \left(\xi \right)=-2\sqrt{A}\cot\;\left(\sqrt{A}\;\xi \right)+\alpha_{0}$$

where

ξ=x+y−4At.

When *A* = 0, the solution takes the form,

62 $$u\left(\xi \right)=\frac{2}{\xi }+\alpha_{0},$$

where

ξ=x+y−4At.

From the above results achieved by the two methods, we can draw the following conclusive remarks:

Remark 1. If we put *A = −* 4*p* where *p* > 0, the results arranged in Eq. (59) are identical to the results (34) and (37) respectively.

Remark 2. If we put A = −4*p* where *p* < 0 then the results arranged in Eq. (61) are identical to the results given in (40).

Remark 3: Result given in (62) is alike to the result given in (52).

## Discussion

From these remarks we have the assessments: The exact solutions of the breaking soliton equation obtained by means of the extended tanh-function method can also be found by the further improved (*G'*/*G*)-expansion method and in appendage some new solutions are obtained.

Recently Parkes (Parkes 2010) showed that the extended tanh-function expansion method proposed by Fan in (Fan 2000) and the basic (*G'*/*G*)-expansion method proposed by Wang et al. in (Wang et al. 2008) are entirely equivalent in as much as they deliver exactly the same set of solutions to a given nonlinear evolution equation. He demonstrated that in many articles in which the basic (*G'*/*G*)-expansion method has been used, the authors claimed that new solutions have been derived but unfortunately these solutions are often erroneous; the alleged the so called new solutions are merely the disguised versions of the previously known solutions. Parkes also claim that the extended tanh-function expansion method delivers solutions in an easy way, alternatively extra effort is necessary when using the basic (*G'*/*G*)-expansion method. But, in this article we have detected that the projected further improved (*G'*/*G*)-expansion method and the extended tanh-function expansion method are not equivalent although the extended tanh-function expansion method and the basic (*G'*/*G*)-expansion method are equivalent. We see that by means of the extended tanh-function we attain merely three solutions of the breaking soliton equation, alternatively through the further improved (*G'*/*G*)-expansion method we obtain twelve solutions of which three solutions are analogous, two are reducible and the rest of the seven solutions cannot be found by the extended tanh-function method. The calculations of the projected method are also easier than the extended tanh-function method as well as the basic (*G'*/*G*)-expansion method.

## Conclusions

A further improved (*G'*/*G*)-expansion method is suggested and applied to the (2 + 1)-dimensional breaking soliton equation. The results obtained by the suggested method have been compared with those obtained by the celebrated extended tanh-function method. From this study, we observe that the further improved (*G'*/*G*)-expansion method and the extended tanh-function method are not equivalent, although El-Wakil (El-Wakil et al. 2010) and Parkes (Parkes 2010) have shown that the basic (*G'*/*G*)-expansion method and the extended tanh-function method are equivalent. We see that all the results obtained by the extended tanh-function are found by the suggested method and in addition some novel solutions are attained. It is evident that obtained solutions are more general and many known solutions are only special case of them. The analysis shows that the proposed method is quite resourceful and practically well suited to be used in finding exact solutions of NLEEs. We expect the suggested method might be applicable to other kinds of NLEEs in mathematical physics and this is our next job.
